# Human milk *vs.* cow-milk based infant formula proteins: structure, digestion and physiological impacts

**DOI:** 10.3389/fnut.2025.1635919

**Published:** 2025-08-18

**Authors:** Lucile Chauvet, Elise Charton, Marion Lemaire, Isabelle Le Huërou-Luron, Amélie Deglaire

**Affiliations:** ^1^STLO, INRAE, L’Institut Agro, Rennes, France; ^2^Institut NuMeCan, INRAE, INSERM, Univ Rennes, Saint Gilles, France; ^3^SODIAAL International, Centre Recherche AND Innovation, Rennes, France

**Keywords:** milk protein, protein structure, non-protein nitrogen, digestion, plasma amino acid, gut microbiota, gut physiology, metabolism

## Abstract

This review examines the differences in protein composition, digestion, and physiological effects on infants between human milk (HM) and infant formula (IF). The World Health Organization recommends exclusive breastfeeding for the first 6 months of life due to the numerous health benefits associated with it. However, when this is not possible, IF is used as an alternative. Differences between HM and IF remain, particularly in terms of protein composition and structure. Further optimization of IF is needed to better mimic HM and provide similar health benefits. Further improving IF formulation requires implementing a promising strategy, which in turn requires a thorough understanding of the mechanisms of protein digestion and amino acid (AA) absorption, as well as the metabolic and physiological effects of protein composition and structure. These are often altered by heat treatment and processing in IF. The main differences in the protein composition and structure of HM and IF are presented, including a synthesis of knowledge on the non-protein nitrogen (NPN) fraction. This fraction is too often neglected in milks, despite accounting for one fifth of the total nitrogen in HM. The influence of the protein composition and structure of HM and IF on the digestion of dietary protein and dietary AA absorption is compared, highlighting the need for data on the postprandial AA profile in infants from well-designed clinical trials. Finally, this review examines the differences in protein composition and digestion between HM and IF that lead to distinct metabolic, physiological and microbial outcomes. Future research should focus on understanding the role of partially digested proteins and the NPN fraction in shaping the infant gut microbiota and overall health.

## 1 Introduction

The health benefits of breastfeeding for infants are globally recognized, and the World Health Organization (WHO) recommends an exclusive breastfeeding until 6 months of age (1). However, the rate of exclusive breastfeeding among infants aged 0–6 months remains low worldwide (48%) (1). Non-breastfed infants are therefore fed infant formula (IF), which aims to mimic as much as possible mature human milk (HM). Thanks to increased knowledge and scientific developments, the composition of IF has been improved over the past decades, in the frame of the European regulation (2). The generic formulation and processing route for producing powder IF is described hereafter (3). IFs, for healthy term infants, are commonly formulated from skimmed cow milk (CM), although other protein sources can be used, such as goat milk or soy protein isolates. However, this will not be covered in the present review. In standard IF, the skimmed milk is enriched with whey proteins (WP) to mimic the average casein:whey protein ratio (40:60) of mature HM and thus cover the regulatory aminogram (2). Free amino acids (AAs) may also be added to standard IF to decrease the IF total protein content as some studies have reported that high protein content in IFs may contribute to infant overweight and obesity (4, 5). Purified whey proteins such as lactoferrin (LF), *α*-lactalbumin, and/or osteopontin can also be used in some specific IFs. Lactose (and sometimes maltodextrin, although not present in HM), fat (usually based on a mixture of vegetable oils, to which milk fat ingredients can be added), minerals and vitamins are used for the IF formulation. The liquid preparation obtained is heat-treated to ensure sanitary safety, the mix is then concentrated usually before or after fat addition and homogenization for emulsion stability (average droplet diameter: 0.1–1 μm). The mix is finally spray dried and packaged (3).

Despite the progress made by industrials in the last decades, discrepancies between HM and IF remain particularly in terms of fine composition and structure, referring here to protein quality, and resulting in different physiological properties in the infant. The present review aims to highlight the differences of composition and structure of proteins between HM and standard IF. The fate of digestion, as well as the metabolic and physiological impacts of HM *vs*. IF will then be addressed.

## 2 Proteins and non-protein nitrogen in human milk *vs.* infant formula

Milk nitrogenous compounds consist of a fraction of protein nitrogen (PN) and, to a lesser extent, a fraction of non-protein nitrogen (NPN). Milk proteins provide bioactive peptides and AAs required for the synthesis of protein contributing to the growth of the infant and the structural and functional development of its organs and tissues, but also for the synthesis of non-protein nitrogen compounds (NPN) (6, 7). Milk NPN fraction, identified as the acid-soluble nitrogen obtained after protein precipitation, consists of more than 10 classes of compounds, the role of which for infant development is still being discussed.

The impact of different IFs and breastfeeding on atopic diseases and growth in pediatric cow’s milk protein allergy, as well as the effects of food processing on allergenicity, have recently been reviewed (8, 9). This topic is not presented here, as the focus is on the impact of the nature of proteins in human milk and cow’s milk-based infant formulas on digestion and gut physiology, which are less documented areas.

### 2.1 Proteins

The HM protein content (0.8–1.2 g/100 mL) is one of the lowest among other mammal milks, including donkey milk. However, it can rise to as much as 6 g/100 mL in some mammal milks, such as sheep’s milk. This correlated with the growth rate of infants, which is one of the lowest for humans and donkeys, and faster for sheep (10). Milk proteins are classified into three major classes: WPs, caseins and mucins (11). HM is a whey-dominant milk with a casein: WPs ratio of 40:60 in mature milk, while CM, the main source of protein and lactose in IFs, is a casein-dominant milk with a casein: WPs ratio of 80:20 (12, 13). Because of these differences, the manufacture of IFs requires the enrichment of their protein fraction in WPs to mimic the AA profile of HM. This enrichment generally results in an average casein: WPs ratio of 40:60 for IFs, ranging from 30:70 to 80:20 (benchmark on 35 IFs available in France made in 2023). Despite this rebalance, the nature of proteins remains different between HM and IFs, resulting in a higher true protein content in IFs than in HM (14, 15).

#### 2.1.1 Whey proteins

WPs have major relevance for the infant development because of their nutritional input and their bioactive functions, but their nature and concentration vary between HM and IFs.

##### 2.1.1.1 *α*-Lactalbumin

α-La has a high nutritional value as mainly composed of essential AAs (63.2% of total AA content). α-La is a protein rich in cysteine (6.5% of residues *vs.* 3.1% in *β*-LG), and also in tryptophan (3.3% AA residues *vs.* 2.5% AA residues in β-LG), an essential AA involved in important metabolic pathways allowing brain maturation and the development of sleep–wake rhythm, through serotonin and melatonin synthesis (16). Human and bovine *α*-La present a similar structure and a high level of homology (~72%) (14), but a few differences in their glycosylation pattern might partly affect their functionality. α-La is the main whey protein in HM, accounting for ~27% of total proteins, while it is only the second most abundant whey protein in IFs, representing ~9.6% of total proteins and being almost three-fold less concentrated than in HM (17, 18). To balance the low-level of *α*-La in standard IFs, and thus of cysteine and tryptophan, it is necessary to increase the true protein content in IF compared to HM or to formulate an *α*-La-enriched IF, thus allowing to have a lower protein content in IF. The addition of free AAs is also another way to balance the low-levels of α-La in standard IFs. The latter strategy might be beneficial for the infant (19–21), however this can be costly and has to be done in agreement with the regulation. In addition, sensory and nutritional consequences of this free AA supplementation should be considered, as free AAs could modify the IF organoleptic properties and may not remain stable along the shelf-life of the IF. Finally, free AA bioavailability is expected to differ from that of AA-bound protein. This should be further examined to evaluate the impact of such strategy. Clinical studies have demonstrated that *α*-LA-enriched IF may promote gastrointestinal tolerance and plasmatic AA concentrations in a similar way to that of HM-fed infants (19, 22–25).

##### 2.1.1.2 *β*-Lactoglobulin

In HM, β-lactoglobulin (β-LG) is totally absent while it is the most abundant WPs in CM accounting for ~50% of total WPs. β-LG is one of the main allergens found in cow’s milk, although infants with allergies are usually sensitive to several proteins found in cow’s milk, such as caseins or *α*-La (26). IgE-mediated food allergy reactions to dairy proteins can evolve during the first years of life. An allergic reaction to *β*-lactoglobulin is commonly reported at birth, before evolving towards caseins and then *α*-La by the end of the first year (27).

##### 2.1.1.3 Lactoferrin

Lactoferrin (LF) is a protein partially resistant to gastrointestinal digestion. Multiple physiological roles have been reported such as antimicrobial, immunomodulatory, anti-inflammatory, bifidogenic, anticarcinogenic, enzymatic and gene regulation activities (17, 28–32). With an average of 0.15 g/100 mL, LF is the second major WP of HM, accounting for ~16% of total proteins (17). LF level in CM is 10 times lower than in HM. Human and bovine LFs have a rather high homology (69%). LF supplementation in IFs has shown some benefits for infants, with a better weight-gain up to 6 month-old, a reduction of some infant diseases (e.g., lower respiratory tract illnesses), and the modulation of the gut microbiota that better mimics HM-fed infant microbiota (24, 33, 34). *In vitro* evaluation of the antiviral activity of LFs showed that the commercial sources of available bovine milk LFs, recombinant LFs and native human/bovine milk LFs influenced immune responses by significantly and variously modulating pro-inflammatory cytokine gene expression (35). The variation in LF bioactivity may be due to differences in processing conditions (e.g., thermal and high-pressure treatments), iron saturation, and purity (35, 36).

##### 2.1.1.4 Immunoglobulins

Immunoglobulins (Igs) are the largest milk proteins. They are the main antimicrobial substances in milk and are divided into four classes: IgAs, IgMs, IgEs and IgGs. Taking all classes together, HM contains far more IgAs than CM. In HM, IgAs make up to almost 90% of total Igs (0.6 g/L in mature HM), while the major class in CM is IgGs (0.6 g/L) (37). However, in IFs the reported levels of secretory IgGs and IgAs are very low (38). Bovine milk immunoglobulins could maintain their structure resisting the temperature up to 75°C for 15 s, albeit losing their antigen-binding efficacy (39). In addition, the technical application for Holder pasteurization further influenced the retention rate of Igs and LF in HM (40).

##### 2.1.1.5 Proteose-peptone

A minor fraction, called “proteose-peptone,” is present in WPs. Although little information exists in HM, the “proteose-peptone” fraction has been detailed in CM. It is a complex heterogeneous mixture of heat-resistant WPs divided in two classes according to their origin. The first class comprises non-hydrophobic, highly soluble fragments derived from proteolysis of the N-terminal region of *β*-casein by bovine plasmin, designated PP5, PP8S and PP8F. The second class consists of a complex heterogeneous group of hydrophobic glycoproteins, the main one being PP3, also known as LP28, and belonging to the fat globule membrane of CM (41). In CM, the “proteose-peptone” fraction accounts for 10% of total WPs. Differences in the “proteose-peptone” fraction composition exist between HM and CM (42). In HM, this fraction contains ~ 45% carbohydrates, whereas in CM it contains only ~ 11%. Regarding IFs, even though its concentration is not well characterized, the “proteose-peptone” fraction is assumed to be present in WP ingredients used for IFs.

##### 2.1.1.6 Glycomacropeptides

WPs used for IF usually derive from cheese making and thus contain a glycomacropeptide (GMP) fraction corresponding to the carboxyl-terminal fragment of casein-*κ* (43–106) cleaved by the action of chymosin used for cheese making. GMP accounts for 9–15% of total protein in IFs (107). Nutritionally, GMP is of little interest for infants as it lacks aromatic AAs (phenylalanine, tryptophan, tyrosine), cysteine and only has a single methionine residue, whereas it is rich in branched-chain AAs (isoleucine, leucine, valine) and threonine. In HM or IFs derived from ideal whey (obtained after skimmed milk microfiltration), GMP is absent. However, it is released by digestive enzymes during the first step of gastric digestion. It has been reported in adults and animal models that GMP may exert some health-promoting activities (108, 109) and modulate microbiota composition and immune system response (22, 110–114), although these effects are not always observed (115). GMP is considered as a bioactive peptide in the literature (108, 109, 116), but there was no evidence of the concentration at which GMP in IFs may have a physiologic impact (115).

#### 2.1.2 Caseins

Caseins are mainly under micellar forms in HM and IFs (90–95%), but human casein micelles are smaller than the bovine ones (30 to 75 nm in HM *vs.* 100 to 200 nm in CM) (117). Bovine casein micelles are composed of four casein species - α_s1_-, α_s2_-, *β*- and *κ*-caseins - with a molar ratio of 4:1:3.5:1.5. However, only three of these are found in HM: α_s1_-, *β*- and κ-caseins, with a molar ratio of 1.5:7:1.5 (13, 118). Although HM and CM both contain β-caseins, they only share ~50% sequence homology and have different numbers of phosphorylation sites (0 to 5 in HM *vs.* 4 to 5 in CM) (118). During gastrointestinal digestion of *β*-caseins, small peptides rich in phosphorylated AA residues, known casein phosphopeptides (CPPs), are formed. These CPPs facilitate the absorption of calcium, zinc and other divalent cations as they are able to keep these cations soluble (119).

A commercial fraction of purified *β*-casein can be added to IFs to increase the β-casein content and achieve the proportion of β-casein present in HM. In 2022, using an *in vitro* digestion model, Huang et al. (120) investigated the effects of supplementing the IF with either β-casein or *α*-LA on digestion. They highlight that increasing the IF content of one of these proteins resulted in a digestion profile that better mimicked HM digestion. However, further research is needed to confirm these preliminary results.

The interest of non-micellar casein and its impact on IF structure, digestive behavior and physiological consequences will be discussed in a dedicated section in this review.

### 2.2 Non protein nitrogen

Very few recent reviews have been carried out to synthesize knowledge on this fraction, a too-often neglected fraction in milks, even though it accounts for one fifth of total N in HM (Table 1). The NPN fraction of milk contains more than 10 classes of compounds: urea, peptides, free AAs, creatine, creatinine, uric and orotic acids, ammonia, carnitine, choline, amino alcohols of phospholipids, amino sugars, nucleic acids and nucleotides, polyamines, low molecular weight peptide hormones and other biologically active compounds such as growth factor (121) (Table 2). Their concentration depends on the mammal species, but also on other parameters such as lactation stage, time of the day, diet, prematurity, etc. (122, 123).

**Table 1:** Concentration of total, protein and non-protein nitrogen, urea nitrogen and free amino acids nitrogen in human milk, cow milk and cow milk-based infant formula (mean ± SD).

Components	Unit	Human milk	Cow milk	CM-based IF
**Total nitrogen**	*mg N/100 mL*	195 ± 46 (57, 63, 77, 86, 259– 266)	531 ± 31 (63, 64, 259, 265, 267–278)	248 ± 34 (62, 64, 227, 263)
**Protein nitrogen**	% TN	80.0	94.1	91.2
**Non-protein nitrogen**	*mg N/100 mL*	39.1 ± 9.2 (57, 63, 259–266)	31.2 ± 9.2 (63, 64, 259, 265, 267–269, 272– 276)	21.8 ± 7.5 (62, 64, 263)
	*% TN*	20.0	5.9	8.8
**Urea**	*mg N/100 mL*	17.6 ± 4.3 (57, 63, 77, 259– 262, 265)	12.5 ± 3.3 (63, 64, 259, 265, 268, 269, 272, 274)	9.4 ± 2.9 (56, 58, internal data)
	*% NPN*	45.0	39.9	42.9
**Free amino acids**	*mg N/100 mL*	5.2 (63, 77, 85, 260, 263, 264, 279–282)	1.5 (63, 64, 265, 278, 281)	1.4 (37, 58, 276, internal data)
	*% NPN*	13.2	4.7	6.3

IF, infant formula; N, nitrogen; TN, total nitrogen; NPN, non-protein nitrogen.

**Table 2:** Concentration of creatine, creatinine, uric acid, orotic acid, glucosamine, sialic acid, carnitine, ammoniac and polyamines in human milk, cow milk and in cow milk-based infant formula (min - max).

mg /100 mL	Human milk	Cow milk	CM-based IF
Creatine	1.53 (0.32–3.7) (121, 140, 277, 278)	2.33 (0.9–3.55) (140, 258, 261)	2.72 (279)
Creatinine	1.25 (0.22–3.5) (121, 140, 254, 277, 278)	0.59 (0.19–1.21) (128, 140, 258, 261)	0.37 (128)
Uric acid	0.73 (0.24–2.2) (121, 140, 253, 272, 274, 277)	0.95 (0.66–1.55) (140, 258, 261, 272, 274)	0.32 (0.3–0.32) (272, 274, 280)
Orotic acid	0 (147, 148, 272, 274)	1.05 (0.84–1.46) (261, 272, 274)	0.42 (0.3–0.6) (272, 274, 280)
Glucosamine	3.6 (1.6–4.7) (121, 140, 141)	12 (± 1.8) (281)	4.85 (± 0.06) (282)
Sialic acid	3.2 (121)	16–19 (283)	12–27 (283)
Carnitine	0.07 (0.04–0.09) (121, 258, 272, 274, 276)	0.28 (0.18–0.36) (128, 258, 272, 274)	0.17 (0.15–0.19) (128, 272, 274)
NH_3_	0.19 (0.16–0.21) (121, 140, 253)	0.74 (0.6–0.88) (140, 261)	0.17 (0.11–0.21) (107)
Polyamines	0.039 (121, 278, 284, 285)	0.0832 (286)	0.007–0.009 (128, 284, 287)
Spermine	0.022 (0.013–0.034)	0.0410 (286)	0.001 (0–0.002) (287)
Spermidine	0.015 (0.009–0.020)	0.0334 (286)	0.004 (287)
Putrescine	0.002 (0.001–0.004)	0.0088 (286)	0.003 (287)

IF, infant formula.

The NPN fraction in HM accounts for approximately 20% of the total N (11.5–25.9%) in HM while it only accounts for approximately 6% of the total N in CM (4.2–9.9%). In CM-based IFs, the NPN content is variable among brands, accounting on average for 9% of the total N (4.9–13.0%) (Table 1).

The variation of the NPN fraction in CM is often attributed to the variation of the urea level. The urea concentration in CM depends on factors related to the cow’s diet, such as dry matter content, crude protein content, percentage of rumen degradable and rumen non-degradable protein, energy/protein ratio, amount of easily digestible carbohydrates, and water intake. It is also influenced by physiological factors such as breed, body weight, mammary gland health, stage of lactation, age and parity (primiparous or multiparous) of cows, and seasonal factors (124, 125). Milk transport and storage time and processing technologies also play a significant role in modulating NPN concentration. The level of NPN increases with transport and storage time. It is also higher in cheese-derived whey due to its production process (hot or cold ripening) than in ideal whey obtained after skim milk microfiltration (126). The levels of NPN in IFs were reported to be highly dependent on the type of whey used. As a matter of facts, demineralized whey can be obtained from several processes such as ion-exchange and electrodialysis, but also processes such as micro- and ultra-filtration. In a study, Donovan and Lönnerdal (127) demonstrated that the level of NPN was the highest in ion-exchange whey, followed by electrodialyzed and ultrafiltered whey. The ultrafiltered whey contained the lowest amount of peptides in the NPN fraction due to the 10 kDa filtration, whereas the ion exchange demineralized whey contained a low amount of highly charged free AAs (Lysine, Glutamic acid, Arginine) (127). In a more recent study on IFs (126), the authors confirmed that demineralized cheese whey contained a higher content of NPN than the demineralized ideal whey.

#### 2.2.1 Urea

Urea is the main NPN component in HM and CM, accounting for around 45 and 40% of this fraction, respectively. This represents 9% of the total N in HM and less than 3% of total N in CM. There is very little data available on the urea content of CM-based IFs (128), but it is estimated to account for around 43% of the NPN fraction and approximately 4% of the total N in IFs. N-urea becomes available after being released by intestinal bacterial ureases, which are expressed by some *Bifidobacterium* species (129, 130). Thus, the composition of the microbiota plays an important role in urea production.

#### 2.2.2 Free amino acids

Milk NPN also contains a fraction of free AAs, which is well-documented in the literature for HM, CM and CM-based IFs. The total free AA content (mg N/100 mL) in HM is 4 times higher than in CM. The free AA fraction reaches 13% of the total NPN fraction in HM and 4.7% in CM. In HM and CM, the free AA fraction is mainly composed of proteinogenic AAs (88 and 74% of total free AAs, respectively) and a small fraction of non-proteinogenic AAs, mainly taurine in HM and carnitine in CM. The most abundant free AA is glutamic acid (39 and 27% of total free AAs in HM and CM, respectively), the second most abundant is glutamine for HM (18%), carnitine for CM (19%), and the third most abundant is taurine in HM (10%) and histidine for CM (13%). Taurine is only the fifth most abundant free AA in CM with a concentration 5 times lower than in HM.

In CM-based IFs, free AAs represent an average of only 6% of the NPN, consisting mainly of proteinogenic AAs (55% of the total free AAs). The non-proteinogenic AA fraction mainly consists of taurine (33% of total free AAs) and carnitine (12% of total free AAs), both of which can be added to IFs. As a result, taurine is the first most abundant free AA (33% of total free AAs), followed by carnitine, serine and glutamic acid, in CM-based IFs. Differences between IF brands are observed because different free AAs (other than taurine) can be added and different protein sources can be used for IF formulation.

Free AAs may be beneficial for infants. They can be directly absorbed and metabolized (121, 131). Glutamine and taurine, for example, which are highly represented in the free AA fraction, play essential roles during infant growth. Glutamine, in particular, is the precursor of non-essential AAs, including proline and arginine, which are produced by the intestinal mucosa (132). Taurine indirectly aids fat absorption by conjugating with bile acids to form bile salts (133, 134). However, studies have shown that taurine supplementation in IF does not modulate fat absorption; rather, it contributes to the development of a healthy microbiota, as taurine-conjugated bile salts are less toxic to *Bifidobacteria* than glycine-conjugated bile salts (135, 136).

#### 2.2.3 Amino sugars

Milk provides N-containing oligosaccharides and amino sugar-containing glycoproteins and glycopeptides. Sialic acids (N-acetyl-neuraminic acid, Neu5Ac in HM and CM; N-gycolylneuraminic acid, Neu-5Gc, in CM), glucosamine and galactosamine are amino sugars present in the NPN fraction (288, 289). Sialic acid is very abundant in HM, with 70 to 83% bound to HM oligosaccharides, 14 to 28% bound to glycoproteins, 2 to 3% in free form, and 0.3% bound to glycolipids (137). Because amino sugar-containing compounds are acid soluble and oligosaccharides are the third most abundant solid component in HM [5 to 15 g/L in mature milk, (138)], nitrogen from amino sugars contributes to a significant portion of the NPN fraction in HM (121). By contrast, oligosaccharides are only present in trace amounts in CM (30 to 60 mg/L) (139). Three studies on HM reported the content of glucosamine (121, 140, 141) and one study reported the content of sialic acid in HM (121). The results showed that glucosamine N accounted for 9.1% of NPN and sialic acid nitrogen for 8.2% of NPN, making them the third and fourth most abundant N compounds in the NPN of HM. Glucosamine and galactosamine contribute to the development of the microbiota, especially *Bifidobacteria*.

#### 2.2.4 Creatine and creatinine

Creatine and creatinine contribute, respectively, 3.9 and 3.2% to the NPN in HM, and 7.5 and 1.9% in CM. In IF, there was insufficient data to determine the contribution of creatine and creatinine to the NPN fractions.

#### 2.2.5 Uric and orotic acids

Uric acid constitutes 1.9% of NPN in HM, 3% in CM, and 1.5% in CM-based IFs. Orotic acid is absent from HM, despite accounting for 3.4% of NPN in CM. There is no data available for orotic acid in CM-based IFs.

#### 2.2.6 Ammonia, nitrite and nitrate

Ammonia contributes 0.5% of the NPN in HM, and 2.4% of NPN in CM. Interestingly, ammonia contributes an average of only 0.8% of NPN in CM-based IFs.

Nitrite accounts for 0.24% of NPN in HM, whereas it is negligible in CM and CM-based IFs, accounting for just 0.0002 and 0.0001%, respectively. In contrast, nitrate is less abundant in HM than in CM and CM-based IFs, accounting for 0.001, 0.17, and 0.09% of NPN, respectively (142).

#### 2.2.7 Polyamines

The contribution of polyamines (spermine, spermidine, putrescine and cadaverine) to the NPN fraction is low in milk (290). Studies have shown that the polyamine concentration is much higher in HM than in IF (0.093–0.140 mg/100 mL HM vs. 0.014 mg/100 mL IF) and intermediate in CM (0.041 mg/100 mL CM) (128, 143, 144). However, only the contribution of N from polyamines to the NPN fraction in HM has been calculated (0.1% of NPN) (134, 143). There is insufficient data on IFs to determine the contribution of polyamines to the NPN fraction, but it is expected to be negligible in comparison to CM.

Despite their low content, polyamines are thought to be necessary for optimal gastrointestinal growth, as they are assumed to contribute to the regulation of cell growth and proliferation (143), and to modulate the composition of the microbiota. This has been observed in neonatal mice fed a polyamine-supplemented IF (145).

#### 2.2.8 Nucleos(t)ides

The concentration of most nucleos(t)ides tends to decrease gradually as lactation progresses, but the decrease is less abrupt in HM than in CM (146–148). Similar compositions are observed in human and bovine colostrum, whereas differences in composition and concentrations are reported in mature milk (141, 143). Although the concentration of ribonucleotides reported in HM varies widely (10 to 200 μmol/L) (148), it appears that HM contains a higher concentration of nucleos(t)ides than CM. Cytidine and adenosine derivatives represent ~25% of the total nucleotides in HM, a higher amount than in CM. In HM, the concentrations of cytidine monophosphate (CMP), adenosine monophosphate (AMP), guanosine monophosphate (GMP), uridine monophosphate (UMP) were found to be 0.076 ± 0.006 mg N/100 mL, 0.105 ± 0.061 mg N/100 mL, 0.024 ± 0.004 mg N/100 mL, 0.032 ± 0.008 mg N/100 mL, respectively (141, 142, 144). GMP, UMP, and inosine monophosphate (IMP) were not detected in CM (140, 142–144). The usual concentration of ribonucleotides (mono- and diphosphate) is about 1 to 2 orders of magnitude higher than that of ribonucleosides (adenosine, guanosine, thymine, uridine, cytidine) in HM (148). In IF, the concentration of ribonucleos(t)ides appears to be a very negligible fraction of NPN, although insufficient data are currently available. Due to the limited data available and the variability of the values reported in the literature, it is difficult to assess the contribution of nitrogen from ribonucleos(t)ides to the NPN fraction in milk. However, nucleotides can be added to IF, which is a common practice.

#### 2.2.9 Amino alcohols

Amino alcohols such as phosphoethanolamine, ethanolamine, phosphoserine, phosphatidylcholine, sphingosin, which mainly come from MFGM, and unesterified choline contribute to the NPN fraction. However, there is very little data on their concentrations in milk available in the literature. In 1989, Atkinson and Lönnerdal (121) estimated that 0.6 to 2 mg N/100 mL could be derived from phosphoethanolamine and phosphatidylcholine, and 0.3 to 0.9 mg N/100 mL from unesterified choline, in HM. Overall, amino alcohols may account for 2–7% of the NPN fraction in HM. There are insufficient data in the literature to evaluate their contribution to NPN in CM or in CM-based IFs.

Overall, although IFs are designed to closely resemble HM in terms of composition, there are still differences in their fine composition and structure. These differences are mainly due to the different milk source (bovine *vs*. human). HM is a bioactive fluid whose composition varies during lactation and depends on the mother’s diet. In contrast, IFs which are formulated to meet the infant’s nutritional needs between 0 and 6 months. IFs have a higher protein content to provide sufficient essential AAs to meet the infant’s nutritional needs, whereas HM provides other bioactive components that are partially lacking in IFs (149).

## 3 Protein digestion and amino acid absorption in infant

At birth, the infant faces many environmental changes, including a change in the way nutrients are delivered. Initially supplied by the placenta during the fetal period, ingested food is destructured throughout the infant’s gastrointestinal tract to allow for nutrient release and absorption, exposing the digestive system to compounds other than those present in the amniotic fluid (150). To meet this challenge, the infant’s organism and metabolism adapt and change during the first 2 years of life. This period is known as the critical period (151) is characterized by the infant’s extreme sensitivity due to the functional immaturity of many tissues and organs. Therefore, it is essential to control the environment to ensure the infant’s optimal growth and metabolic development.

### 3.1 Digestive specificities in infants

Maturation of digestive functions begins early *in utero*, with enteral feeding possible as early as 29 weeks of amenorrhea (152), but the digestive system is still immature at birth, which affects the infant’s ability to digest and absorb nutrients, including proteins.

#### 3.1.1 Protein digestion

Protein digestion starts in the gastric phase, where proteolysis is carried out by pepsin, secreted as pepsinogen and activated autocatalytically at a pH below 4 (153). Despite active acid secretion from birth, the infant’s gastric mucosa is still highly susceptible to acidity during the first two to 3 weeks of life, and thus prostaglandins (such as PGE2) stimulate mucus and bicarbonate production, inhibit acid secretion and increase mucosal hydrophobicity (154). As a result, the gastric pH is between 6.0 and 6.5 immediately after feeding and does not reach pH 5.0 until 80 min after feeding due to the high buffering capacity of the milk diet (HM and IF) (155). At this pH, the rate of conversion of pepsinogen to pepsin is low and the activity of pepsin is probably reduced, as its pH optimum has been reported to be 2.0 for the hydrolysis of globular proteins (156) and may be different for the unstructured proteins that are caseins. The level of pepsin activity at 4 weeks postpartum has been reported to be 18% of that in adults (152) and to reach 100% only at 2 years of age (125, 148, 151). Gastric proteolysis in infants is thus incomplete as compared to adults. A previous study (153) showed that the type of diet (HM *vs.* IF) has no effect on pepsin production during infancy. *In vivo*, gastric proteolysis in nine full-term neonates averaged 15% of ingested protein (157), while *in vitro* gastric proteolysis of 5 to 15% of ingested protein is often reported for a static, semi-dynamic, or dynamic model of term neonates (120, 152–155). Overall, this suggests that a large proportion of dietary proteins enter the intestinal compartment partially or non-hydrolyzed. Several studies have reported that the rate of gastric emptying is influenced by the type of diet. HM is assumed to have a faster gastric emptying than IF, with a T_1/2_ of 36 to 48 min in preterm and term infants for HM (158) and a T_1/2_ of 65 to 78 min in preterm and term infants for IF (159). Two recent *in vivo* studies also confirmed faster gastric emptying with HM than with IF (157, 160). Several factors have been reported to influence gastric emptying, including bolus volume, caloric density, lipid quality, and protein type and structure (161).

The pancreas plays an important role for intestinal proteolysis through its secretory activity of major proteases (162). Pancreatic juice contains inactive proteases in the form of zymogens and contribute to pH increase of the acidic gastric chyme thanks to sodium bicarbonate. The activation of these zymogens is the result of cascade mechanisms. Trypsin is the most important pancreatic enzyme, accounting for 20% (w/v) of the total protein in pancreatic juice. Its activity is detected as early as the 16^th^ week of amenorrhea and increases throughout the fetal period, reaching 90% of that of adults at birth (43, 163). In infants, chymotrypsin activity is estimated to be 50 to 60% of that of children over 2 years of age (43). Other pancreatic proteases complete the action of trypsin, such as chymotrypsin, elastases and carboxypeptidases, the latter one being the only exopeptidases among the pancreatic proteases. The intestinal protein hydrolysis is completed by the action of the brush border peptidases. The addition of brush border peptidases to pancreatic proteases in an *in vitro* digestion model raised the degree of proteolysis from 57 to 74% (44). At the end of the intestinal proteolysis, the luminal nitrogen is composed of small peptides of two to three AA residues (~60 to 70%), free AAs (~25 to 30%), and a small fraction consisting of large peptides and undigested proteins (45). The undigested or unabsorbable nitrogen fraction is then available for fermentation by the colonic microbiota. Fermented nitrogen can either be reused by the bacteria, excreted in the feces or, to a lesser extent, absorbed by the host in the form of ammonia and free AAs (46, 47).

#### 3.1.2 Absorption of proteolysis products

A large proportion of peptides is absorbed in the jejunum, while free AAs are assumed to be mainly absorbed in the ileum (48). Compared with the other two segments, the absorption of free AAs and their derivatives from bacterial fermentation in the colon is low. The complex structure of the intestinal epithelium, whose development begins in the fetus and ends shortly after birth, greatly increases the exchange surface area. Before entering the portal vein, di- and tripeptides may undergo a final stage of proteolysis in the cytoplasm of brush-border enterocytes. Free AAs are absorbed using several transport systems present in the apical and/or basolateral membranes of enterocytes: active (Na^+^-dependent) transporters and facilitated transport by simple diffusion across the membrane, while di- and tri-peptides are absorbed by a H + -dependent transporter (PepT1) (49). The absorption of AAs and peptides is developmentally regulated and influenced by diet, hormones and growth factors. The transport systems are present at birth, and the transport rates of peptides and most AAs tend to decrease with age (from birth to weaning), although the extent of these changes varies widely among individuals (43). Studies suggested that the high dietary levels of protein or AA lead to the transcriptional activation of the PepT1 gene, resulting in the upregulation of peptide and AA transport (50–52).

#### 3.1.3 Metabolic fate of dietary amino acids

From a quantitative perspective, the appearance of AAs in portal blood depends primarily on the composition and quantity of ingested proteins, as well as their digestibility. The main factors that determine the kinetics of AA appearance are the physicochemical nature of the proteins and how quickly they can transit and/or be digested through the digestive tract (53–55). A large proportion of AAs are used as they are, or can be transaminated for use as other AAs in protein synthesis. Another fraction enters specific metabolic pathways that convert AAs into non-protein nitrogen compounds. A final fraction can be catabolized to produce energy.

Free AAs first circulate through the splanchnic zone before reaching the peripheral circulation. In piglets, it has been reported that approximately 27% of total dietary nitrogen is retained in the splanchnic area following a meal (56). However, splanchnic extraction of AAs varies widely among AAs. For instance, up to 80% of dietary threonine and around 50% of dietary lysine are absorbed by the splanchnic area in piglets (57–59). Studies in adult humans have reported splanchnic extraction rates of up to 96% for glutamic acid, 69% for alanine, and 64% for glutamine. In contrast, the rates were much lower for arginine (38%), phenylalanine (29%) and leucine (21%), expressed as a proportion of enteral intake (60). Several parameters influence the metabolic fate of AAs in the splanchnic zone, such as the tissues with discrepancies between liver and gastrointestinal tissues, the tissue cell characteristics, and the molecular form of AAs (56). After extraction of AAs by the splanchnic zone, the circulating pool of AAs consists of dietary AAs that have escaped splanchnic extraction, AAs from transamination of dietary AAs in the liver, and AAs from proteolysis of endogenous proteins. These circulating AAs are metabolized by peripheral organs. In piglets, 42% of dietary nitrogen retention occurred in the peripheral zone [including 31% in the muscle and 6% in the skin (56)].

As a growing organism, the neonate has a positive nitrogen balance (anabolic predominance), with protein losses inversely related to gestational age (61). However, in 2008, Kalhan and Bier (62) reported that the protein losses expressed as a function of metabolic weight (weight^0.75^) are not significantly different from those of adults, whereas they are significantly higher when related to body weight. In seven-day-old piglets, the rate of protein accretion was very high and dependent on enteral intake (63). Focusing on specific AAs, the rate of glutamine and phenylalanine appearance in circulating blood, expressed on a body weight basis, was higher in infants than in healthy adults, reflecting higher energy expenditure in the infant (62, 64). It was also demonstrated that a negative relationship between glutamine turnover and the irreversible oxidation of protein (urea synthesis) existed, thus suggesting that glutamine has an important role as a nitrogen source for other synthetic processes and accretion of body proteins in the newborn (64). Finally, tryptophan is an AA of importance for infants because it is a limiting AA in food, being present in low concentration in IFs. Tryptophan released after protein hydrolysis is mainly used for protein biosynthesis. The remaining fraction of free tryptophan is metabolized by the host in the intestine, liver and brain, or by the colonic microbiota (65). The kynurenine pathway in liver is the most significant degradation pathway of the tryptophan unused for protein synthesis (>90% of tryptophan in excess available) (66). It is metabolized to quinolinic acid, which in turn is converted to niacin (vitamin B3). Tryptophan is important for infants because it is also converted in the pineal gland to the neurotransmitters, serotonin and melatonin, which play an important role in sleep regulation. Interestingly, the transport of tryptophan across the blood–brain barrier is in competition with the transport of other large neutral AAs (LNAAs), namely histidine, isoleucine, leucine, methionine, phenylalanine, tyrosine and valine. As a result, a strong correlation between brain tryptophan concentration, brain serotonin concentration and plasma tryptophan/LNAA ratio has been demonstrated (67–69).

### 3.2 Impact of protein composition and structure on digestion of HM *vs.* IF

HM is a dynamic fluid that serves as the biological standard for infant nutrition, providing the essential nutrients and thousands of bioactive molecules that play critical roles in protecting against infection and inflammation, contributing to immune maturation, supporting organ development, and promoting healthy microbial colonization. Unlike IF, which targets 0- to 6-month-old infants, HM composition adapts during lactation to meet the specific needs of the developing infant, which vary by stage of lactation, and between term and preterm infants (70).

HM serves as the reference for IF formulation. IFs require numerous ingredients (up to fifty for some IFs) and production steps involving several heat treatments, either on the raw material during ingredient manufacturing or during IF production (3). The formulation and processing route of IFs can differ between industrials, contributing to variability of IFs in their protein composition and potential ingredient interactions. This can affect digestion kinetics and physiological effects (71, 72). With respect to proteins, several factors such as phosphorylation, size, charge, tertiary structure, AA content and glycosylation have been identified as influencing protein degradation (131). Casein and WPs, both in HM and IFs, differ chemically and structurally, influencing their gastric behavior and digestive sensitivity. Interestingly, the ratio of casein to WPs influences gastric emptying and the degree of proteolysis (55, 73). Gastric emptying in infants aged three to 12 months was faster after the ingestion of HM or WP-dominant IF than casein-dominant IF (158), as casein coagulates near its isoelectric point (4.6). In contrast, unaggregated WPs remain soluble at the acidic pH of the infant stomach. In 2020, Halabi et al. (74) demonstrated that the addition of LF within IF induced partial casein micelle disintegration even before heating. Some studies have also explored the effect of casein mineralization and organization on gastric behavior and digestive kinetics (75–77), showing that the lower the mineralization, the faster the proteolysis. These results may explain the variability in gastric half-emptying time reported by different studies for IFs, as the mineralization levels of casein in IFs on the market is highly variable (4 to 12 mmol micellar Ca per 10 g casein (78);) and can also explain why the gastric emptying is slower for IF than for HM, due to the lower mineralisation level of HM caseins [~3.2 mmol micellar Ca per 10 g casein; (79)]. This can induce a different pattern of protein coagulation in the stomach, in addition to the different structure of human and bovine casein micelles (80–82) and the different size of fat droplets in HM and IF. Furthermore, there are different variants of *β*-casein (A1 and A2) in cow’s milk and IFs. Milk based on A2 β-casein is available on some markets. It has been reported that A2 β-casein cow’s milk has a different casein micelle size and different curd formation properties. This could potentially result in reduced gut discomfort related to milk (83). However, whether this remains true within the IF context requires further investigation.

Thermal treatments of proteins, such as those employed in ingredient and IF processing, are known to alter both protein structure and gastric behavior. Heat treatments increase the resistance of casein to gastric hydrolysis by forming casein/WP aggregates (84). A recent work also showed that the heat-induced denaturation of LF within IF significantly increased its susceptibility to hydrolysis (85). Changes in the surface properties of casein micelles, after WP binding, enhanced casein hydrolysis within IF (85) and increased the pH at which casein coagulates from ~4.9 to ~5.4 (86). The casein micelle organization was proved to be strongly dependent on the *β*-LG and LF contents in IF and on the heating temperature (74). The dairy protein ingredients used in IF formulations may be dry or liquid, depending on the manufacturer. A main factor influencing protein denaturation and aggregation during the initial processing of the liquid raw materials is the heat intensity (87–89). For dairy protein ingredients, the production process (serum, concentrate or isolate) appears to affect the rate of protein denaturation and aggregation, resulting in differences in digestibility (90).

Focusing on WPs, several studies have shown that *β*-LG in its native form remains intact in the stomach. Heat treatment can either accelerate its proteolysis in the intestine due to protein chain unfolding phenomenon (91–95), or reduce its digestibility by forming compact, aggregated WPs that hinder enzyme access to cleavage sites (96, 97). A study investigating the impact of heat treatment on proteins within IF revealed that IF containing *α*-La and LF preserved a higher proportion of native WPs than IF containing β-LG for high heat treatments (90°C/15 s, 75°C/15 min) (98). In its native form, LF has been shown to be resistant to pepsin, enabling it to exert its full bioactivity in the gut. However, the pasteurization of HM results in the partial gastric hydrolysis of LF, as demonstrated *in vitro* under infant digestive conditions (99, 100).

The Maillard reaction (glycation), favored by the heat treatment that occurs during IF production and storage due to the presence of primary amines and of lactose (a reducing sugar), can also affect protein digestibility directly or indirectly by sterically hindering the cleavage sites of digestive enzymes (90). In 2020, Zenker et al. (101) showed that high levels of glycation in IFs increase the size of peptides during digestion, indicating reduced proteolytic efficiency. Reduced glycation levels in IFs may enhance the gastrointestinal comfort of infants, as indicated by the clinical trial of Sheng et al. (102). However, the latter result is confounded by the different nature of the prebiotics administered in the various IFs. In addition, Maillard reaction products, such as carboxymethyl lysine, may also affect intestinal physiology (103, 104).

A recent *in vitro* and *in vivo* study on HM and IF (164) showed that the microstructure of the digesta differs between HM and IF, and that gastric proteolysis of *α*-La and casein is significantly lower for HM than for IF. In addition, it was demonstrated that the quality (structure and composition) of dairy protein ingredients within IF formulation significantly influenced the microstructure of the IFs. These differences were found to modulate proteolysis kinetics as well as the breakdown of the emulsion during the early gastric phase (126), highlighting the importance of considering the quality of protein ingredients in IF manufacturing. Whenever possible, IFs should be designed to closely resemble HM, including its digestive behavior (3, 105).

The true ileal digestibility (TID) of HM *vs*. IF was recently measured in Yucatan mini-piglets as a model for human infants (106). It was shown that the TID of total nitrogen was lower for HM than for IF due to the greater proportion of NPN in HM. NPN remains largely unabsorbed and is transferred to microbiota. As previously discussed in this review, this may have physiological relevance. Additionally, the TID of seven AAs differed significantly, particularly for lysine, phenylalanine, threonine, valine, alanine, proline and serine. The digestible indispensable AA score (DIAAS) of IF was lower than that of HM (83 *vs*. 101). Moughan et al. highlighted that the current FAO (2013) recommendations for AA requirements for infants may require revision, as the recommended AA concentrations were not corrected for hydrolysis time and digestibility. This resulted in lower values for leucine, lysine and threonine (more than 16% difference) and histidine and tryptophan (more than 30% difference).

### 3.3 Impact of protein composition and structure on absorption of dietary amino acids of HM *vs.* IF

The kinetics of AA appearance in plasma can be significantly impacted by several parameters, with the gastric emptying rate being the most important factor (55, 165). Previous studies on the casein fraction in IFs have shown that the degree of casein mineralization affects their supramolecular organization, which in turn influences their behavior in the stomach (76, 78). In other dairy matrices, it has been confirmed that the supramolecular organization of casein influences the gastric emptying rate and, subsequently, postprandial AA kinetics (77, 166). Another factor influencing the appearance of AA in the plasma is the rate of protein digestion, which in turn is modulated by protein structure. Specifically, the structure of WPs is easily modified in IFs, due to the numerous heat treatments. *In vivo* studies comparing postprandial AA concentrations in neonatal piglets fed native or denatured WP solutions have shown that higher plasma levels of essential AAs are observed within the first 60 min with native WP than with denatured WP (167). Similarly, in rodents fed diets containing 40% IF with either native or heat-denatured WPs (168), the consumption of native WPs resulted in higher plasma levels of total AAs than heat-denatured WPs.

A study in neonatal mini-piglets investigated how the composition and structure of protein ingredients within IFs affects plasma AA kinetics and concentrations (107). Although no difference in plasma AA kinetics was observed, both preprandial and postprandial AA concentrations in the plasma were modulated by the quality of the protein within the IFs. Interestingly, the whey origin within the IFs (either cheese or ideal whey) was found to modify AA homeostasis, resulting in increased plasma total and essential AA concentrations in piglets fed cheese whey IFs compared to ideal whey IFs. In line with previous studies (169), the importance of using cheese whey to increase plasma threonine concentration was emphasized, given that the cheese whey contains a significant amount of GMP, a threonine-rich peptide (107). Several other studies focusing on the casein/WP ratio in IFs found that an increased WP content in IFs was associated with higher plasma threonine concentrations, which were directly related to the AA profile of the predominant proteins (23, 170, 171). Several authors have suggested that increased threonine intake may be related to the limited ability of infants to eliminate threonine, given that it leads to higher plasma threonine levels (107, 169, 170, 172, 173). Interestingly, formula-fed term infants have been reported to have higher baseline and postprandial plasma concentrations of threonine, urea and valine, than breastfed term infants (174).

Several studies have attempted to compare the levels of AAs in the plasma of infants fed HM *vs.* formula. Most emphasized that the different protein profile in IFs compared to HM impacts the plasma concentration of AAs, as well as urea and other compounds, in preterm (169, 170, 172) and term (23, 170, 175–177) infants. However, only one study was conducted under presumed isonitrogenous conditions. This study showed that the postprandial plasma concentration of essential AAs was ~18% lower in HM-fed infants than in formula-fed infants. This suggests that the nature and structure of proteins may play a modulating role in AA absorption (178), in addition to the molecular form of nitrogen. It is likely that nitrogen was present in HM in lower proportions as true proteins than in IF.

Comparable data on HM and IF is lacking in this area due to differences in the design of the studies carried out, the nitrogen content of the matrices under study, the absence of a dietary adaptation period prior to sampling, the lack of sampling kinetics and imprecision regarding the timing of blood sampling in relation to the last meal. Furthermore, plasma AA concentrations, as measured in the systemic circulation, are influenced by first-pass extraction from splanchnic tissues, as well as by protein turnover and AA metabolism in the whole organism. These factors could differ between matrices (i.e., within IFs and HM).

## 4 Infant diet impact on microbiota, intestinal and brain development

Early nutrition plays an essential role in programming adult health and disease, including metabolic, cardiovascular, and immune diseases, as well as programming food preferences and eating behaviors (179, 180). The health benefits provided by HM are not yet fully realized with IF feeding. Therefore, improving the functional effects of IFs is an important goal in order to reduce the gap in terms of physiological and metabolic health between breastfed and formula-fed infants.

In the short term, HM has been associated with a lower risk of neonatal mortality. HM appears to be protective against sudden infant death syndrome as compared to IF (181). The reduced risk prevalence was a function of the breastfeeding rate, with a greater reduction for exclusive or predominant breastfeeding. Furthermore, there is convincing evidence that HM reduces the risk of developing diarrhea (0–5 years) (181–183) and acute otitis media (0–2 years) (183–185). In the long term, HM-fed infants may be at a lower risk (− 22%) of being overweight or obese in adulthood compared to formula-fed infant (4, 186–190). Furthermore, HM may prevent type II diabetes in adulthood compared to IF (181, 191–193).

The infant development initiated *in utero* continues from birth up to 2–3 years of age. Many changes occur during the early postnatal period, especially regarding intestinal development through morphological changes, functional maturation such as microbiota establishment (194, 195) and brain development (synaptogenesis and myelination) (196). Environmental factors such as infant nutrition (HM *vs*. IF) are essential determinants of the postnatal development, reported to modulate the gut microbiota (197–199), and thus the global microbiota-gut-brain axis (199, 200).

### 4.1 Microbiota, a key determinant of the infant health through its role on the gut brain-axis

Gut microbiota represents a complex bacterial ecosystem that colonizes the digestive tract. Itferments indigestible nutrients (such as HMOs or urea) and produces various metabolites including SCFAs (201). The early development of microbiota is under the influence of many parameters like the mode of delivery, the maternal genetic, the use of antibiotics and the environment as well as the maternal and infant nutrition (15, 180, 202, 203).

A lower fecal *α*-diversity was observed in HM-fed infants compared to formula-fed infants at 40 days of age, the difference being further reduced at 6 months of age (204–207). Indeed, gut microbiota abundance and evenness increased in HM-fed infants after 3 months of age unlike that in formula-fed infants for whom these indexes were already high at 40 days of age gut microbiota (207). A lower α-diversity was also measured in different intestinal segments (ileum, colon and rectum) in 3 weeks-old HM-fed piglets than in formula-fed piglets (198, 206), suggesting a specific role of HM components. Actinomycetota (formerly Actinobacteria) is the major phyla in gut microbiota of 3- and 6-month-old HM-fed and formula-fed infants, representing over 42 to 74% of the total phyla. The microbiota of HM-fed infants is characterized by a lower relative abundance of Bacillota (formerly Firmicutes) than that of formula-fed infants (205, 207), but Bacillota remains the second major phylum in 6-month-old HM-fed infants. Dissimilarities also exist at the genus level. *Bifidobacterium* (including *Bifidobacterium* spp., *Bifidobacterium breve, Bifidobacterium infantis, Bifidobacterium longum*) is frequently described as the dominant genus in the fecal content of 0- to 9-month-old HM-fed infants unlike that in formula-fed infants (203, 207–209), although some studies reported no difference (210, 211).

Besides HMOs, lipids and lactose (193, 212–214), it has been demonstrated that proteins and NPN are able to modulate the microbiota composition (15). Firstly, the impact of the nitrogenous fraction on microbiota shaping is frequently associated to proteins having immunomodulatory properties such as LF, lysozyme or Igs, but can also be due to other proteins and to NPN. Regarding immunological proteins, LF decreases the iron availability for bacteria due to its bacteriostatic property and is able to decrease *E. coli*, *Pseudomonas aeruginosa* and *Candida albicans* (215). Moreover, lysozyme can hydrolyze the peptidoglycan polymers found in the cell walls of bacteria, thereby lysing Gram-positive bacteria. However, it can also act synergistically with LF, contributing to the degradation of Gram-negative bacteria (216). Other studies have suggested that the undigested proteins from IF could also contribute, albeit to a lesser extent, to modulating the gut microbiota. Supplementating IF with *α*-La and GMP has been shown to increase the relative abundance of Clostridiaceae, Enterobacteriaceae and *Streptococcus* in preterm piglets after 19 days of feeding (217), whereas no difference was observed in six-month-old term infants (218).

Other nitrogenous compounds that could influence the microbiota include NPN, particularly urea, for which a role has been reported. Indeed, some bacteria possess urease genes, including certain *Bifidobacterium* species (e.g., *B. longum* subsp. Infantis); meaning that urea could act as a growth factor for these species (130, 219). Supplementation the IF with nucleotides was shown to decrease the ratio of *Bacteroides*-, *Porphyromonas*-, *Prevotella*- to *Bifidobacterium*-species in the fecal microbiota of 20-week-old infants (220).

### 4.2 Impact of infant diet on gut immune and barrier functions and brain development and their relationships with gut microbiota

Food in direct contact with the intestinal epithelium plays a recognized role in the maturation of the intestinal barrier, immune and endocrine functions. The structural and functional development of the gut depends on the type of the diet consumed (HM *vs*. IF). Intestinal growth is enhanced by bioactive components of HM, such as growth factors, absent or present in low amount in IF (194, 221).

It is acknowledged that the postnatal development of intestinal permeability in humans and porcine models follows a bell curve (222–224). During the first weeks of life, higher permeability is most commonly reported in HM-fed infants as compared to formula-fed infants (225), and in HM-fed and sow’s milk-fed piglets as compared to formula-fed piglets (199, 226). This has been associated with a reduced expression of genes involved in tight junction proteins in HM-fed piglets compared to formula-fed ones (199, 227). However, conflicting data have been observed in animal models, such as no change in jejunal or ileal permeability and/or increased expression of tight junction proteins in sow’s milk- *vs.* formula-fed piglets (206, 226, 228), and no change in the lactulose/mannitol ratio in breastfed infants (229–231). Such discrepancies may be related to the specificity of epithelial permeability in the intestinal segments studied in animal models, which cannot easily be recapitulated by an overall measurement such as the lactulose/mannitol ratio in infants. High intestinal permeability can lead to an increased passage of molecules across the epithelium, thereby promoting immune system development and tolerance to commensal bacteria and dietary antigens (226). Accordingly, HM-induced enhancement of the mucosal immune system has been reported in breastfed infants (232–234) and in piglets fed only HM (199). Fecal calprotectin content, a valuable marker of the state of intestinal mucosal inflammatory infiltration, was higher in HM-fed infants compared to formula-fed infants during the first weeks of life (225, 231, 235–237).

Several immunological factors, such as LF and IGs (IgG, IgA, IgM), which are more abundant in HM than in IF (38), anti-inflammatory cytokines (such as TGF-*β* and IL-10), pro-inflammatory cytokines [IL-1b, IL-6, IL-8, IL-12, TNF-*α*, IFN-*γ*; (15)] and other minor proteins present in HM but not in IF (17), are likely to contribute to the immune system boost in addition to dietary-induced changes in microbiota composition. The importance of the Bacillota phylum in inducing a pre-weaning peak in intestinal inflammatory markers has been demonstrated in rodents and piglets. Accordingly, our study comparing HM-fed and IF-fed piglets supports the relationship between microbiota and the mucosal immune system maturation. Several significant positive correlations were observed between *Anaerovibrio, Mitsuokella* and *Veillonella* genera belonging to Veillonellaceae family (Bacillota phylum) and genes encoding anti- and pro-inflammatory cytokines (IL-10, IL-10Ra, SOCS3, CCL2, IL-1bR, IL-8, TNF-α) and cellular signaling (ICAM1, MYD88) (199). This boost of the mucosal immune system has been shown to be essential for both immune ontogeny and regulation of susceptibility to immunopathologies later in life (199, 238). Moreover, in infants, positive correlations between fecal calprotectin excretion and colonization by some taxa of the Bacillota phylum support the role of bacteria in maturation of the intestinal immune system (239). The quality of the protein ingredients in IFs has also been reported to moderately affect the gut physiology and microbiota of three-week-old mini-piglets used as a model of human infants (115). This suggests that the quality of WP and casein (structure and composition) may mediate some of the physiological properties of IFs.

Similar to the gut, brain development begins *in utero* and continues throughout the first years of life. The postnatal period is crucial for the development of the central nervous system (240). While some steps in the development of neurons that begin at birth, such as visual and auditory processing, are rapidly developed, others, such as synaptogenesis and synaptic refinement, take longer to be fully developed (240, 241). These processes occur from birth to 3 years of age. It has been established that HM and IF have different effects on brain development and function. A large observational study (>17,000 healthy infants) has provided strong evidence that HM is more beneficial for optimal infant neurodevelopment than IF (242). It has also been demonstrated that HM is associated with higher myelination than IF at 2 years of age. Better myelination resulted in superior language and motor function in HM-fed infants, which is consistent with the superior cognitive performance observed in HM-fed infants compared to IF-fed infants during the first 6 months of life (243–245). Later in life, better cognitive function was observed in 10-19-year-olds who were breastfed as infants, compared to those who were formula-fed during infancy (246). However, several other environmental factors may affect child development. Supporting these clinical observations in infants, HM and IF diets were found to induce different genes expression profiles related to blood–brain barrier, endocrine and immune functions, neurosynaptogenesis, and metabolite levels in various brain regions, particularly the hypothalamus and hippocampus, in piglets (199).

The difference in brain development profiles between infants fed HM and IF can mainly be explained by differences in food composition related to components of the milk fat globule membrane (MFGM), such as polar lipids (244, 247) and to polyunsaturated fatty acids (248–251). Furthermore, a recent study in piglets demonstrated that the composition and structure of WPs and caseins did not affect the expression of several genes associated with hypothalamic development (115).

## 5 Conclusion

HM is a biofluid that provides the necessary nutrients to support infant growth. It contains many components that have been shown to affect metabolism, gut physiology, and the development of infant gut microbiota. Proteins are among the most important of these components for optimal growth. When formulating IF, the quality of the protein ingredient must be considered, although difficulties remain due to the different protein profiles of bovine and human milk. The nutritional value of milk from other mammals requires further investigation. There is a lack of data on the causal relationship between the protein composition and quality of IF and the plasma AA profile, highlighting the need for convincing studies. Furthermore, the quantity of HM compounds that reach the colon, and the impact of partially digested proteins (e.g., HM-derived peptides) on the composition of the microbiota, must be investigated to fully comprehend the role of the HM nitrogenous fraction in shaping the infant gut microbiota. In particular, the NPN fraction may be important, despite not yet being considered in current IF formulations.
